# Reducing Recurrence in Equine Corneolimbal SCC: Outcomes of Adjunctive Cisplatin Biodegradable Bead Therapy

**DOI:** 10.3390/vetsci13010076

**Published:** 2026-01-12

**Authors:** Amy Dagenais, Tristan Juette, Marie-Odile Benoit-Biancamano, Maria Vanore

**Affiliations:** 1 Centre Hospitalier Universitaire Vétérinaire, Department of Clinical Sciences, Faculty of Veterinary Medicine, Université de Montréal, 3200 Sicotte St., St-Hyacinthe, QC J2S 8H5, Canada; amy.dagenais@umontreal.ca; 2 Centre de Diagnostic Vétérinaire de l’Université de Montréal, Department of Pathology and Microbiology, Faculty of Veterinary Medicine, Université de Montréal, 3200 Sicotte St., St-Hyacinthe, QC J2S 8H5, Canada; marie-odile.benoit-biancamano@umontreal.ca; 3 Faculty of Veterinary Medicine, Université de Montréal, 3200 Sicotte St., St-Hyacinthe, QC J2S 8H5, Canada

**Keywords:** cisplatin, squamous cell carcinoma, eye, adjunctive therapy, ophthalmology, horse

## Abstract

(1) Context: Squamous cell carcinoma (SCC) is the most prevalent ocular neoplasm in horses, with a reported 45% recurrence rate when managed by surgery alone. (2) Objective: To evaluate the effect of adjunctive cisplatin biodegradable beads (CBBs) on recurrence rates of equine corneolimbal squamous cell carcinoma (SCC) and to document associated adverse effects. (3) Methods: Seventeen cases of histopathologically confirmed corneolimbal SCC in horses were retrospectively identified. Masses were surgically excised by keratectomy and/or conjunctivectomy under general anesthesia, followed by implantation of CBBs beneath conjunctival flaps at approximately 1 cm intervals around the excised area. Horses were monitored through owner follow-ups for up to five years postoperatively. (4) Results: Three horses (17.64%) experienced local mass recurrence within one year of treatment, and one horse (8.33%) relapsed approximately two years post-treatment. Minor local adverse effects—including chemosis (36%), hyperemia (64%), localized yellow discoloration (55%), and granular tissue formation (36%)—resolved within one to two months after surgery. Two horses developed bead-associated uveitis requiring closer ophthalmic monitoring. Vision was preserved in all but one relapsing case. (5) Conclusions: These findings suggest that adjunctive use of cisplatin biodegradable beads during surgical excision of equine corneolimbal SCC may significantly reduce recurrence rates and is generally well tolerated.

## Simple Summary

Squamous cell carcinoma is a common ocular neoplasm in horses. Though the cancerous mass can sometimes be removed by surgery, it often grows back. In fact, the mass returns in about half of the treated horses. This study reviewed medical records from 17 horses with this neoplasm to see if combining surgery with a specific chemotherapy implant (on the eye itself) could help prevent mass recurrence. All horses were monitored for 1–5 years after treatment. Only 4 of the 17 horses (~24%) had the neoplasm return during the follow-up period. In terms of adverse effects, most were mild and temporary, like irritation or inflammation of the eye. Only two horses had adverse effects that needed closer follow-up. Overall, these findings suggest that using this specific chemotherapy implant as an adjunct treatment with surgery is generally safe and may decrease the chances of this cancer returning in horses.

## 1. Introduction

Squamous cell carcinoma (SCC) is a common presenting complaint in equine veterinary ophthalmology, typically characterized by a pink, exophytic mass affecting the eye or ocular adnexa. As the most prevalent ocular neoplasm in horses, reported distributions indicate that ocular SCC most frequently involves the eyelids (28%), nictitating membrane (26%), cornea (19%), and the limbus or bulbar conjunctiva (25%), although prevalence by site varies among studies [1]. Ocular SCC is locally invasive but slow to metastasize [2,3]. When metastases occur, they typically involve the regional lymph nodes and salivary glands, while distal metastases most often affect the lungs [4].

Although the pathogenesis of ocular SCC remains incompletely understood, certain breeds are predisposed due to genetic and environmental factors. Horses with limited periocular pigmentation (e.g., Hafflingers, Appaloosas), draft or outdoor working breeds with increased UV exposure (e.g., Belgian Draft horses), and carriers of a loss-of-function mutation in the DDB2 gene are at increased risk of UV-induced oncogenic damage [2,3,5]. Early surgical intervention is critical to preserving vision, maintaining comfort, and improving prognosis.

However, recurrence following surgical excision alone is common. In a seminal study of 49 cases across 43 horses with ocular SCC, King et al. reported a 45% recurrence with surgery alone and a significant reduction in recurrence when surgery was combined with adjunctive therapy [6]. Common adjuvant modalities include radiotherapy, cryosurgery and intraoperative laser ablation [7,8]. Chemotherapeutic options such as intermittent mitomycin C protocols administered over several weeks may also be used, though owner compliance and patient tolerance can be limiting factors [7,8].

Cisplatin biodegradable beads (CBBs) represent a slow-release chemotherapeutic delivery system currently used to treat cutaneous neoplasms in horses [9]. These beads are composed of an inorganic biopolymer matrix of calcium and dextran sulfate, allowing the sustained local diffusion of 7% cisplatin over approximately 1 month. With an efficiency of 1 mg/cm^3^, the 3 mm beads are typically spaced at 1 cm intervals within subcutaneous pouches created during surgery [10]. Interestingly, loss of the DDB2 gene—a known predisposing factor to equine ocular SCC—renders cancer cells more sensitive to genotoxic agents such as cisplatin [11,12]. Despite the demonstrated efficacy of CBBs in SCC and various other cutaneous neoplasms in Equidae [9,13], their potential use in ocular SCC has not yet been characterized.

To address this gap, we aimed to evaluate the recurrence rates and complications associated with adjunctive CBB therapy in equine corneolimbal SCC. In this retrospective study of 17 histopathologically confirmed cases, CBBs were implanted around the excision margins and anchored to the sclera beneath a conjunctival flap using absorbable sutures. Owner follow-ups ranging from one to five years revealed local recurrence in four horses, suggesting markedly lower early and late recurrence rates compared with surgery alone. Minor postoperative adverse effects—including discharge, ocular pain, conjunctival inflammation, and local discoloration—resolved within one to two months after surgery. Two horses developed severe uveitis requiring more intensive ophthalmic monitoring. Vision was preserved in all but one relapsing case. Overall, these findings suggest that CBBs may represent a useful addition to the limited therapeutic options currently available for equine corneolimbal SCC. Further investigation is warranted to refine this approach and improve clinical outcomes for affected horses.

## 2. Materials and Methods

### 2.1. Inclusion Criteria

A retrospective study was conducted with client-owned horses admitted for corneolimbal SCC at the Centre hospitalier universitaire vétérinaire (CHUV), Université de Montréal, between 2009 and 2025. Horses were eligible for this study if they (a) were otherwise healthy, (b) had a histopathologically confirmed diagnosis of corneolimbal SCC, and (c) were treated surgically by keratectomy/conjuctivectomy with adjunctive CBB implantation. Horses with a prior history of keratectomy or conjunctivectomy at the same location were excluded. Only cases with documented owner follow-up for at least one year post-surgery were retained.

Seventeen cases across 16 adult horses of various breeds were identified, with admissions between 2009 and 2023. Telephone follow-ups were conducted by the CHUV ophthalmology service with owners at 1, 3, 6, and 12 months postoperatively, and annually thereafter for up to 5 years (when applicable), to obtain information on patient mass recurrence. Additional patient and case information was obtained from CHUV medical records, including breed, age, sex, coat color, weight, histopathological findings, and postoperative complications.

### 2.2. Ophthalmic Examination

A complete ophthalmic examination was performed by a board-certified veterinary ophthalmologist at the CHUV using a slit lamp (Kowa SL-17^®^; Innova, Toronto, ON, Canada) and graded according to a modified Hackett-McDonald scoring system. When ocular integrity and patient compliance permitted, intraocular pressure was measured using rebound tonometry (TonoVet^®^; Icare Finland Oy, Vantaa, Finland). Depending on the diagnostic plan selected by the owner, ocular ultrasonography was performed using a 12 MHz ophthalmic probe (Sonomed Escalon; Lake Success, NY, USA). Photographs were also taken for patient medical files (Genesis-D Kowa, Innova; Toronto, ON, Canada).

### 2.3. SCC Surgical Excision and CBB Placement

Premedication, induction, maintenance, and recovery were managed by a board-certified veterinary anesthesiologist. For loco-regional anesthesia, lidocaine or bupivacaine was administered both locally and topically (“splash block”) following anesthesiologist recommendations. Horses were positioned in lateral recumbency, and the surgical site was aseptically prepared and draped for ocular surgery.

**Figure 1:**
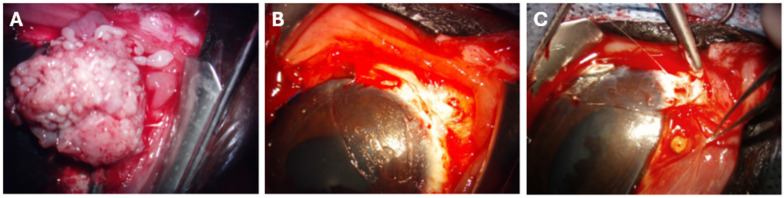
Representative placement of a CBB during the surgical excision of equine corneolimbal SCC of the left eye. The horse was anesthetized by a board-certified veterinary anesthesiologist and prepared for surgery in lateral recumbency. (**A**) The corneolimbal mass to be excised, before intervention. (**B**) The debulked limbal region, post-mass excision. (**C**) Retraction of a tissular pouch cut into the bulbar conjunctiva in the debulked limbal region, revealing a CBB before suturing.

Superficial or deep lamellar keratectomy and/or conjunctivectomy were performed under an operative microscope (ZEISS OMPI MDO XY S5; Jena, Germany) by a board-certified veterinary ophthalmologist (Figure 1A). Eyelid retractors or a lateral canthotomy were used to improve exposure of the surgical field. With continuous corneal irrigation using sterile saline, the bulbar conjunctiva was incised using Westcott scissors and the corneal plane using a Beaver 64 blade and a Martinez lamellar corneal dissector (MicroSurgical Technology; Redmond, WA, USA). The corneolimbal mass was dissected in either a superficial or deep plane until complete excision was achieved, with minimal corneal and conjunctival margins of approximately 2 mm and 3 mm, respectively. Hemostasis was achieved using sterile cotton swabs and 2.5% phenylephrine solution (Alcon; Fort Worth, TX, USA).

After complete tumor excision, the adjacent bulbar conjunctiva was dissected and mobilized toward the limbus (Figure 1B). CBBs (3 mm, 7% cisplatin; Royer Biomedical, Inc., Frederick, MD, USA) were inserted under the conjunctiva at approximately 1 cm intervals to span the length of the excised area (Figure 1C) and secured to the sclera with interrupted 6-0 PDS sutures (Ethicon; Sommerville, NJ, USA). Handling and exposure followed standard protocols for injectable cisplatin [14,15]. A subpalpebral catheter (MILA; Hebron, KY, USA) was placed for postoperative drug administration before completing the procedure.

The excised tissues were fixed in 10% neutral buffered formalin or Davidson’s fixative. All samples were submitted to the Centre de diagnostic vétérinaire de l’Université de Montréal (CDVUM) for routine processing and hematoxylin–phloxine–saffron staining, followed by evaluation by board-certified veterinary pathologists to confirm the diagnosis, assess tumor infiltration, and evaluate surgical margins.

### 2.4. Postoperative Medical Management

Horses were hospitalized for a minimum of three days after surgery for observation and to initiate topical therapy. Topical ophthalmic treatments were continued until corneal wound healing and included tobramycin (0.2 mL, q4-8h; Sandoz; Boucherville, QC, Canada), atropine sulfate (0.2 mL of 1%, q12h until mydriasis, then q24h; Alcon; Fort Worth, TX, USA), and autologous serum (q2-8h) administered via a subpalpebral catheter placed in the upper or lower eyelid. Systemic anti-inflammatory therapy consisted of flunixin meglumine (1.1 mg/kg PO q12h for seven days, then q24h for seven days; Banamine^®^), with serum creatinine monitored every three days and treatment adjusted as needed. Additional analgesics were added on a case-by-case basis (notably 13 mcg/kg/h butorphanol for 24 h post-surgery [16]; Torbugesic^®^ or 20 mg/kg acetaminophen q12h for three days [17]; Plus Pharma, CA, USA). After discharge, horses were re-evaluated by the CHUV ophthalmology service or by their referring veterinarian for long-term follow-up, depending on owner preference and logistical constraints.

### 2.5. Statistical Analyses

Statistical analyses were performed using R software version 4.5.1 [18] with the “car” and “pwr” packages. To compare the observed recurrence rate distributions with the expected prevalence of 45% reported for surgery alone (6), exact binomial tests were employed. To assess the effect of explanatory variables on early (less than one year post-treatment), late (greater than one year post-treatment), and overall recurrence rates, generalized linear models (logistic regression) were employed, specifying a binomial error distribution [19]. Model results were obtained using likelihood ratio tests [20]. A *p*-value < 0.05 was considered significant.

## 3. Results

### 3.1. Animals

To assess the efficacy and safety of CBBs as an adjunctive therapy for equine corneolimbal SCC surgical excision, 17 cases from 16 horses were evaluated postoperatively. The cohort is presented in Table 1, comprising Haflingers (n = 9), Quarter Horses (n = 3), one Appaloosa (n = 1), one Belgian (n = 1), one Hanoverian (n = 1), and one Thoroughbred (n = 1). The mean age was 11.7 ± 5.4 years, and the cohort included 10 males and 6 females. Coat color (10 light, 4 dark, 2 unspecified) and weight (mean = 542.9 ± 118.1 kg; one unspecified) are also listed for each horse. Tumor invasiveness (5 in situ, 10 invasive, 2 unspecified) and completeness of excision (7 complete, 6 incomplete, 4 unspecified) are reported for each case.

### 3.2. Local SCC Recurrence Rates

Owners were contacted for updates on local mass recurrence (Table 2), with the last follow-up ranging from one to five years after surgical excision and bead implantation. Recurrence was categorized as early (<1 year), late (>1 year), or overall, based on owner reports. Of the 17 cases, three (17.6%; 95% CI = 3.8–43.4%) experienced early recurrence, and one case (8.33%; 95% CI = 0.2–38.5%) experienced late recurrence approximately two years post-treatment, for a total of four relapsing cases (23.5%; 95% CI = 6.8–49.9%). Only one recurrent mass was submitted for histopathological confirmation. Both early (*p* = 0.027) and late (*p* = 0.016) recurrence rates were significantly lower than the reported 45% rate for surgery alone (6), suggesting that adjunctive use of CBBs may reduce the risk of corneolimbal SCC recurrence in horses. However, the trend for the overall recurrence rate was not found to be significant (*p* = 0.090), possibly due to added variability from the combination of early and late periods for an insufficient number of total replicates.

Possible explanatory variables for differences in recurrence rate were evaluated, including tumor-related factors (infiltration, completeness of excision) and patient factors (age, sex, coat color, and weight). None of the examined variables had a significant effect on any of the three recurrence categories (Table 3).

### 3.3. General Adverse Effects After Bead Insertion

To characterize the adverse effects associated with adjunctive CBB therapy, each medical record was reviewed for explicit documentation of bead-related or unanticipated postoperative complications (Table 4). Of the 17 cases, three had inaccessible daily notes with no mention of abnormalities, and three were considered notable and are described separately. In turn, 11 cases were used for the description of general adverse effects.

**Figure 2:**
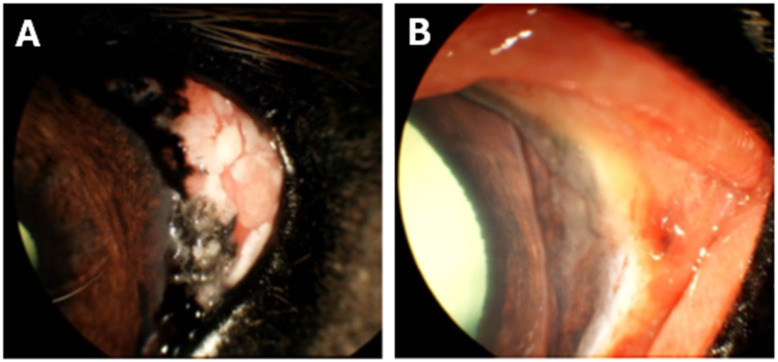
Representative adverse effects post-insertion of CBBs on the bulbar conjunctiva. Horse 8 was diagnosed with corneolimbal SCC of the left eye, which was surgically excised and adjunctively treated with CBBs. (**A**) The corneolimbal mass before surgical management. (**B**) In the weeks following treatment, yellow discoloration of the bulbar conjunctiva, conjunctivitis, hyperemia, and chemosis were noted around the bead insertion site. All clinical signs dissipated within the month.

**Figure 3:**
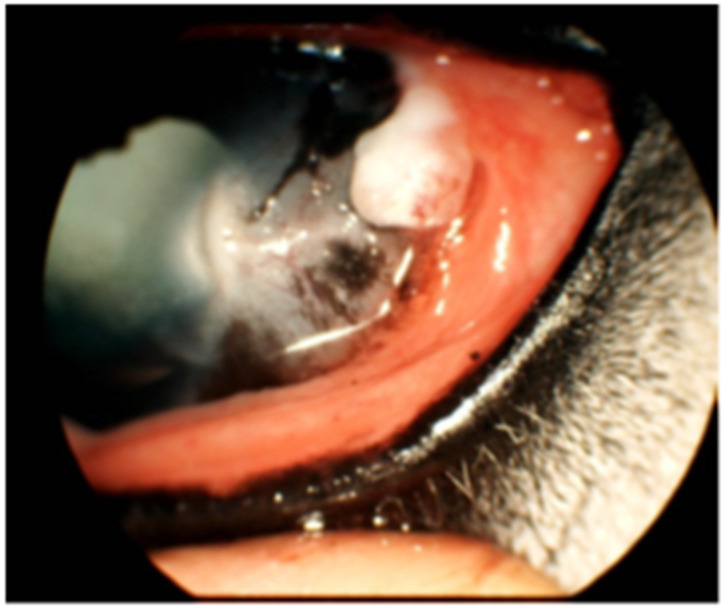
Representative conjunctival granuloma following perilimbal insertion of CBBs at the lateral canthus of the left eye. Horse 5 was diagnosed with corneolimbal SCC, which was surgically excised and adjunctively treated with CBBs. A conjunctival granulomatous proliferation developed at the bead insertion site and persisted in the following weeks.

The most frequently documented finding was ocular discharge, ranging from serous to mucopurulent (7/11 cases). Signs of ocular discomfort, including blepharospasm, rubbing, and head shaking, were noted in 6/11 cases. Conjunctivitis (4/11), conjunctival hyperemia (7/11), and conjunctival chemosis (4/11) were also common around the bead insertion sites (Figure 2). Localized yellow discoloration of the cornea or bulbar conjunctiva (6/11; Figure 2) and granulation tissue formation (4/11; Figure 3) occurred near the beads. Low-grade uveitis, attributed to a localized bead-induced inflammatory response, was documented in one case.

All minor adverse effects were medically managed as needed by a board-certified veterinary ophthalmologist or referring veterinarian, with resolution within one to two months post-surgery. No systemic adverse effects were reported, and vision was maintained in all eyes. These findings suggest that CBBs placed within the bulbar conjunctiva are generally well tolerated for the treatment of corneolimbal SCC in horses.

### 3.4. Notable Patient Courses After Bead Insertion

Among the documented postoperative adverse effects, three cases warranted detailed description. Although most reactions were mild, two horses developed severe uveitis with granuloma formations.

Horse 11 was medically managed for two months post-treatment, presenting with blepharospasm, abundant mucopurulent discharge, conjunctival hyperemia, fibrin in the anterior chamber, and hyphema. The granuloma from the initial surgery was later excised and histopathologically confirmed. Horse 12 required surgical removal of the beads and surrounding inflamed conjunctiva one week after insertion due to severe mucopurulent discharge, chemosis, and hyperemia. Despite bead removal, uveitis persisted for three months, presenting with blepharospasm, hyperemia, diffuse corneal edema, corneal yellowing, dyscoria, hypopyon, anterior and posterior synechiae, and a non-visualizable lens and fundus. With medical management, the eye returned to baseline, and vision was restored. These cases emphasize the importance of close ophthalmic follow-up when using CBBs for corneolimbal SCC in horses.

**Figure 4:**
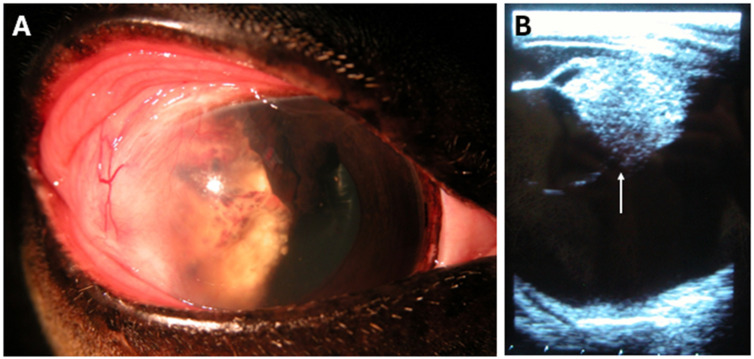
Early recurrence case removed from adverse effect considerations due to confounding timeline. Horse 7 was diagnosed with corneolimbal SCC of the right eye, which was surgically excised and adjunctively treated with CBBs. Upon re-evaluation 3 weeks postoperatively, (**A**) a yellow mass with vascular proliferation was visible in the lateral part of the anterior chamber. Lateral perilimbal corneal fibrosis was consistent with the keratectomy site for removal of the initial proliferative SCC mass. (**B**) Ocular ultrasonography performed at 12 MHz revealed a hyperechogenic mass located posterior to the iris and ciliary body (arrow), consistent with intraocular SCC proliferation. Enucleation was subsequently performed.

One early recurrence case (horse 7) was excluded from the adverse events dataset due to confounding factors (Figure 4). Upon re-evaluation three weeks postoperatively, this horse presented with diffuse corneal edema, neovascularization, abundant yellow discharge, and a non-visualizable fundus. Following further investigation, the owner elected enucleation, which revealed SCC invasion of the anterior chamber. Because complications may have resulted from SCC progression rather than CBB placement, these findings were not included in the adverse event analysis. Horse 7 was the only patient in the study that permanently lost vision.

## 4. Discussion

The purpose of this retrospective study was to assess the potential efficacy and tolerance of adjunctive CBBs in reducing recurrence of equine corneolimbal SCC. The corneolimbal form, which represents over one-quarter of all equine ocular SCC cases [2,21], is particularly challenging to manage because of its limited potential for complete resection compared with lesions of the nictitating membrane or eyelids. Adjunctive therapies that are well tolerated by the cornea and bulbar conjunctiva are therefore essential to minimize recurrence.

As random sampling was not possible given the retrospective design, the study population consisted largely of breeds predisposed to ocular SCC. Although not representative of the broader provincial horse population, this cohort reflects the high-risk group most likely to develop and present for clinical care of this disease. Similarly, the cohort composition mirrored previously reported trends in breed and age distribution [2,3,6,22]. However, contrary to previous reports, no significant influence of potentially confounding factors such as age, sex, coat color, or weight (used as a proxy for breed) was detected. Sample size calculations indicated that approximately 222 individuals would be required to detect a statistically significant relationship with a power of 0.80, which may explain why subtle associations were not observed with the limited sample size available.

Although specific recurrence rates for equine corneolimbal SCC have not been widely reported, we based our hypothesis on the conservative estimate of 45% recurrence for ocular SCC treated by surgical excision alone [6]. Some clinical resources cite recurrence rates as high as 80% following surgery alone [23]. In contrast, adjunctive CBB therapy significantly reduced early (17.6%) and late (8.3%) recurrence rates, comparable to the 25% recurrence reported with adjunctive radiation therapy [6]. It is worth noting that only one recurrent mass was examined histopathologically, which could have artificially inflated the apparent recurrence rate if some growths represented inflammatory or granulomatous lesions rather than true neoplasia. However, the overall recurrence rate trend was nonsignificant (*p* = 0.090), possibly due to the limited sample size available. In turn, these findings support the efficacy of CBBs as a clinically useful adjunctive approach for lowering equine corneolimbal SCC recurrence rates, though further investigations with larger sample sizes are warranted.

With regard to local adverse effects, additional investigation is needed to better understand the mechanisms underlying the observed clinical signs. Conjunctival changes—including conjunctivitis, hyperemia, and chemosis—may initially result from surgical trauma, whereas their persistence and gradual improvement over the first postoperative month could be related to mild cytotoxic irritation during cisplatin release. The diffusion of the yellow-tinted cisplatin through conjunctival vessels into the cornea, conjunctiva, or underlying iris may also explain transient discoloration. Conjunctival granulomas similar to those seen here have been previously described following carbon dioxide photoablation use as adjunctive therapy for ocular SCC [8], attributed to reparative or immune responses to conjunctival trauma [24,25], or to conjunctival injury more generally [26]. Whether granuloma formation in this context was primarily related to the conjunctival flap or the beads themselves remains unclear. Interestingly, no cataracts were reported secondary to CBB usage. Since other adjunctive therapies—particularly periocular radiation—are well recognized for inducing cataracts [27,28], CBBs may offer an advantage in preserving visual comfort and function.

Two horses developed severe uveitis following surgery and adjunctive therapy, one of which required revision surgery to remove the beads. Although systemic anti-inflammatories were administered during hospitalization and 5–7 days after discharge, more intensive topical therapy might be required to mitigate excessive inflammatory responses. Indeed, similar severe inflammation has been reported with the use of other ocular implants in horses, such as intravitreal devices for the continuous release of cyclosporine A [29]. Given that other ocular implants have also been shown to increase the risk of surgical site infection in horses [29,30], the use of products aimed at altering or maintaining the ocular microbiota may be warranted. The use of CBBs should therefore be monitored closely by a veterinarian during the month of cisplatin release to detect and manage potential secondary uveitis or other mild complications. Further studies should also aim to determine optimal bead spacing and placement parameters to balance therapeutic efficacy with the risk of adverse effects.

The main limitations of this study are inherent to its retrospective nature, including variability in data quality and completeness, lack of randomization, treatment heterogeneity, and potential follow-up bias. Interpretation was also constrained by the small number of surgical cases and the absence of a control group of horses treated with surgery alone. However, for ethical reasons, withholding adjunctive therapy from owners seeking to minimize recurrence would be difficult to justify. Similarly, reliance on owner reports and limited histopathological analysis of recurrent masses made it difficult to ascertain true recurrence rates post-treatment. From an applicability standpoint, the use of CBBs has historically been limited or unavailable in some regions, which may affect widespread adoption in clinical practice [31,32].

## 5. Conclusions

In summary, this study suggests that cisplatin biodegradable beads are a relatively safe and effective adjunctive treatment to reduce the risk of corneolimbal SCC recurrence in horses. With only local minor and no systemic adverse effects, and with preservation of vision in most cases, CBBs provided continuous, localized delivery of cisplatin without the need for owner manipulation. Overall, these findings underscore the value of continued research into innovative, practical treatment options for equine corneolimbal SCC.

## Figures and Tables

**Table 1:** Signalment and tumor features of the study cohort. Signalment data extracted from the CHUV medical file database for horses presenting with corneolimbal SCC managed by surgery and adjunctive CBB therapy from 2009 to 2025. Breed, age (years), sex (M/F), coat color (light/dark) and weight (kg) are listed for each horse. Missing data points are represented by NA.

Horse	Breed	Age (Years)	Sex	Coat Color	Weight (kg)	Tumor Infiltration	Tumor Excision
1	Appaloosa	16	M	Light	500	NA	Incomplete
2	Belgian	10	M	Light	930	In situ	Complete
3	Haflinger	8	F	Light	410	In situ	Complete
4	Haflinger	4	M	Light	545	Invasive	NA
5	Haflinger	11	M	Light	555	Invasive	Incomplete
6	Haflinger	10	M	Dark	515	Invasive	NA
7	Haflinger	10	F	Light	498	NA	NA
8	Haflinger	20	M	Dark	459	In situ	Complete
9	Haflinger	11	F	Light	455	Invasive	Incomplete
10	Haflinger	13	F	Light	516	Invasive	Complete
11	Haflinger	10	F	Dark	508	Invasive	Complete
12	Hanoverian	7	M	Light	607	Invasive	Complete
13	Quarter horse	27	F	NA	550	Invasive	NA
14	Quarter horse	11	M	Dark	NA	Invasive	Complete
15	Quarter horse	10	M	NA	579	Invasive	Incomplete
16	Thoroughbred	9	M	Light	517	In situ	Incomplete

**Table 2:** Cases of local mass recurrence. Local mass recurrence following surgical excision and CBB therapy, as extracted from the CHUV medical file database and associated client communications log for horses in the study cohort. Time of recurrence (early/late) and histopathological confirmation of corneolimbal SCC are specified for each horse. Cases marked with an “X” correspond to horses that developed recurrence.

Horse	Early Recurrence (<1 Year)	Late Recurrence (>1 Year)	Histopathological Confirmation
4	X		No
5		X	No
6	X		No
7	X		Yes

**Table 3:** Potential explanatory variables for differential recurrence rates. The potential impacts of tumor features (tumor infiltration, surgical excision) and patient signalment (age, sex, coat color, weight) on recurrence rate were analyzed using generalized linear models, specifying a binomial distribution of the dependent variable. *p*-values are specified for each explanatory variable for early, late, and overall recurrence. *p* < 0.05 was considered significant.

Explanatory Variable	Early Recurrence	Late Recurrence	Overall Recurrence
*Tumor infiltration*	0.183	0.327	0.997
*Surgical excision*	1.000	0.255	0.200
*Age*	0.076	0.626	0.127
*Sex*	0.937	0.355	0.615
*Coat color*	0.774	0.383	0.930
*Weight*	0.677	0.988	0.776

**Table 4:** Prevalence of ocular adverse effects. Clinical signs following surgical excision and CBB therapy, as extracted from the CHUV medical file database and associated client communications log for horses in the study cohort. The number of presenting horses is specified for each clinical sign, as well as the representative percentage in parentheses.

Clinical Signs	Number of Presenting Horses (Percentage in Parentheses)
Ocular discomfort	6/11 (55%)
Discharge	7/11 (64%)
Conjunctivitis	4/11 (36%)
Chemosis	4/11 (36%)
Hyperemia	7/11 (64%)
Local yellow discoloration	6/11 (55%)
Granuloma	4/11 (36%)
Light uveitis	1/11 (11%)

## Data Availability

The original contributions presented in this study are included in the article. Further inquiries can be directed to the corresponding author.

## Abbreviations

The following abbreviations are used in this manuscript:

CBB	Cisplatin biodegradable bead
SCC	Squamous cell carcinoma
CHUV	Centre hospitalier universitaire vétérinaire
CDVUM	Centre de diagnostic vétérinaire de l’Université de Montréal
